# Core Content, Competencies, and Accreditation in US Global Health Fellowships: A Survey of Leaders’ Perspectives

**DOI:** 10.4269/ajtmh.24-0377

**Published:** 2024-11-12

**Authors:** Thomas F. Siegert, Sophia P. Gladding, Patricia F. Walker, Janis P. Tupesis, Andrew P. Steenhoff, Ashti A. Doobay-Persaud, Elizabeth D. Barnett, John W. Sanders, Brett R. Hendel-Paterson

**Affiliations:** ^1^ Department of Pediatrics, Division of Medicine-Pediatrics, The Warren Alpert Medical School at Brown University, Providence, Rhode Island;; ^2^ Yukon-Kuskokwim Health Corporation, Bethel, Arkansas;; ^3^ Department of Medicine, University of Minnesota, Minneapolis, Minnesota;; ^4^ HealthPartners Institute, Bloomington, Minnesota;; ^5^ HealthPartners Travel and Tropical Medicine Center, St. Paul, Minnesota;; ^6^ University of Wisconsin School of Medicine and Public Health, Madison, Wisconsin;; ^7^ Division of Infectious Disease & Global Health Center, Children’s Hospital of Philadelphia, Philadelphia, Pennsylvania;; ^8^ Departments of Medicine and Medical Education, Feinberg School of Medicine, Northwestern University, Chicago, Illinois;; ^9^ Section of Pediatric Infectious Diseases, Boston Medical Center, Department of Pediatrics, Boston University Chobanian & Avedisian School of Medicine, Boston, Massachusetts;; ^10^ Section on Infectious Diseases, Wake Forest University School of Medicine, Winston-Salem, North Carolina;; ^11^ HealthPartners Regions Hospital, St. Paul, Minnesota

## Abstract

The number of global health (GH) fellowships in the United States has increased over the past two decades. However, there are currently no standard requirements, shared core content, or widespread systems of accreditation. With the growth in programs, it is appropriate to consider these issues. We conducted a national survey to understand GH fellowship leaders’ perspectives on the existence of core content and competencies and on the need for accreditation, including by the Accreditation Council of Graduate Medical Education (ACGME). We sent survey invitations to 123 fellowship leaders. Forty-five completed the survey (37%), representing seven specialties. Eighty-nine percent of respondents indicated that there is important core content for fellows to learn regardless of specialty; 30% indicated that accreditation would be “very” or “extremely” beneficial, whereas 21% indicated that it would be “not at all” beneficial. When asked what form of accreditation would result in training the most competent GH practitioners, 35% indicated that accreditation is unnecessary. Of those selecting a form of accreditation, the largest proportion (21%) selected accreditation from a professional society; 52% “disagreed” or “strongly disagreed” that ACGME accreditation is needed. More than 65% indicated that loss of training flexibility, funding restrictions, and increased administrative and fellow funding burdens are “very” or “extremely” important barriers. These results suggest that broad agreement on important core content exists across specialties, with a lack of consensus about the need for accreditation. More discussion with stakeholders, including international partners, is needed to understand their perceptions and build consensus before pursuing fellowship accreditation.

## INTRODUCTION

Over the past two decades, the number of global health (GH) fellowships offered across medical specialties in the United States has grown steadily, with 122 programs currently listed in the Global Health Fellowship Database.^1^^,^^2^ This growth reflects increasing interest from medical trainees^1^^,^^3^^,^^4^; the growing importance of GH training to address the globalization of diseases and pervasiveness of health disparities^3^; and the expansion of the discipline, now an area of practice within most clinical specialties. Although there is not a widely accepted, single definition of GH, a common definition is “an area for study, research and practice that places a priority on improving health and achieving equity in health for all people worldwide.”^5^ This definition reflects the widening focus of GH from its origins in tropical medicine and infectious disease to include social determinants of health, health disparities, health equity, advocacy, and care of increasingly mobile populations around the world.^5^

Current GH fellowship programs reflect the breadth and diversity of the discipline across multiple specialties. These postgraduate programs vary in core content covered (the knowledge, skills, and attitudes needed to practice GH), and decisions about educational content made within specialties and programs draw from a variety of sources for core content.^6^^–^^8^ Programs vary in core competencies (the observable abilities of trainees in activities integrating knowledge, skills, and attitudes),^9^ with several specialties developing specialty-specific core GH competencies.^10^^–^^12^ Programs vary in their requirements and practices, although best practices and ethical guidelines have been created in specific areas, such as predeparture preparation, postreturn debriefing, health and safety, and international partnerships.^13^^–^^15^ Programs also vary widely in structure from fellowships focusing primarily on GH training to programs combining GH with other subspecialty training.^1^^,^^16^ They vary in length from several months to several years, and they also vary in the amount and type of international or local–global experience (domestic community or field experience with clinical and nonclinical experiences emphasizing topics such as public health, socioeconomic and political sciences, and medical sciences)^8^ included.^1^^,^^16^

With the field of GH maturing and the continuing growth in GH fellowships, questions related to the lack of standardization of core content, competencies, and program requirements across fellowships increase in importance. Namely, does variability in fellowship programs lead to variability in the competencies that graduates achieve? Would standardized core content and competencies, possibly across specialties, result in better-trained GH practitioners?

Would standardization of program requirements and practices reduce the potential negative impacts on trainees,^17^^,^^18^ hosting institutions,^19^^,^^20^ and the communities being served,^19^^,^^21^^,^^22^ which may result from the uneven uptake of best practices by programs?^16^

One approach to standardization across programs is through fellowship accreditation in which an external body provides formal oversight of adherence to defined standards. Although accreditation can establish minimum program standards, it may also have negative impacts, such as increased programmatic burdens and restrictions. There is currently no widespread accreditation of GH fellowship programs in the United States. In general, fellowships can be accredited by the Accreditation Council of Graduate Medical Education (ACGME),^23^ the most recognized accrediting body in the United States, which leads to subspecialty certification from the American Board of Medical Specialties (ABMS). Alternatively, fellowships can be accredited by a specialty-specific “focused practice,”^24^ by a professional society such as the Academic Pediatric Association (APA), or not at all. At present, two specialties have accreditation or endorsement processes for GH fellowships. Since 2022, pediatric GH fellowships can be accredited by APA,^25^ and emergency medicine has developed an endorsement process for GH fellowships through the Society of Academic Emergency Medicine (SAEM).^26^ Individual trainees can document their knowledge through Certificate of Knowledge tests offered by professional societies, such as the American Society of Tropical Medicine and Hygiene (ASTMH)^27^ and the International Society of Travel Medicine.^28^ Trainees can also pursue advanced degrees, such as a Master of Public Health, or concentrations in GH across graduate programs.

Given the growth in GH fellowships across specialties, the lack of standardized core content and competencies, and the potential benefits of standardization through accreditation, ASTMH convened a national committee of GH educators to explore the feasibility of developing an accredited GH subspecialty. In the fall of 2019, the Global Medicine Exploratory Committee (GMEC) was formed with 11 members representing a range of specialties (infectious disease, internal medicine, pediatrics, family medicine, emergency medicine, and immigrant and refugee health) and a range of roles in GH education, which included a resident physician trainee. A key aspect of the committee’s work and the purpose of our study were 1) to understand United States-based GH fellowship leaders’ perspectives on whether core content and competencies exist in GH that are relevant across specialties and 2) to understand these leaders’ perceptions of the need for some type of accreditation, including ACGME accreditation, which would be required for a formal subspecialty recognition. Although previous studies have surveyed GH fellows^29^ and single specialties^16^ about their perceptions of accreditation, we found no previous multispecialty studies of GH fellowship leaders’ perceptions of accreditation.

## MATERIALS AND METHODS

### Study design and population.

We conducted a cross-sectional survey study of GH fellowship leaders. The target population was current clinical fellowship program leaders (e.g., fellowship program directors and associate program directors) in the United States, with GH fellowships defined as formal postresidency training focused primarily on GH. The study targeted fellowship leaders in primary specialties (internal medicine, pediatrics, psychiatry, surgery, family medicine, obstetrics and gynecology, emergency medicine, and multispecialty fellowships including these specialties) as the committee had determined that these specialties would be the initial focus of the potential multispecialty GH fellowship. We created a list of program leader contacts, all publicly available, using four methods: 1) review of the online Global Health Fellowship Database,^2^ which maintains a list of fellowships; 2) a literature review of United States-based GH fellowships; 3) an internet search for United States-based GH fellowships; and 4) review by the committee members of the program contact list and updated contacts known through professional relationships.

### Survey development.

Global Medicine Exploratory Committee members with expertise in GH education and survey design developed the survey informed by a literature review of articles related to GH fellowships and accreditation. All committee members then reviewed and refined the survey. Finally, three GH education experts outside of the committee reviewed the survey and provided feedback, which was used to further refine the survey. The survey consisted of 24 questions with closed-ended questions (dichotomous, Likert, and Likert-type questions using a five-point scale) and open-ended questions related to 1) the existence and importance of GH core content and competencies across specialties, 2) the potential benefits of and barriers to accreditation, and 3) demographics and fellowship program characteristics (Supplemental Appendix).

### Survey administration.

We sent the survey via REDCap^30^ hosted at the University of Minnesota. The survey invitation described the purpose of the survey, informed participants that all responses were confidential, and included the survey web link. It also included a request for recipients that did not have a current role in GH fellowship education to provide the current contact for their program if possible. We then sent surveys to these new contacts. The survey was open from March to June 2022. We sent up to three reminder e-mails to nonrespondents.

## STATISTICAL ANALYSES

We calculated descriptive statistics (frequencies and percentages) for the closed-ended questions. We used SPSS v. 25 (IBM Corp., Armonk, NY) for all statistical analysis. We conducted qualitative content analysis^31^ to analyze the two open-ended prompts: “Please describe why you selected the form of accreditation you indicated” and “Please include any additional comments you would like to share about the benefits of, need for, barriers to, or possible negative impacts of offering an ACGME accredited fellowship in global health.” One author (T. F. Siegert) read the written comments and created a set of initial codes. Two authors (T. F. Siegert and S. P. Gladding) then met, and they reviewed and refined the codes. The authors then reread and independently coded the written comments using the refined codes, and they met to compare their coding and discussed any disagreements until consensus was reached. The authors then sorted the codes related to reasons for supporting a specific form of accreditation from the codes related to potential impacts of accreditation, and they further sorted those codes for positive and negative impacts of accreditation. The authors then grouped related codes describing the potential negative impacts of accreditation into broader categories. Finally, the authors selected illustrative quotes. The authors selected quotes addressing ACGME accreditation specifically and quotes addressing accreditation generally for each potential impact of accreditation to which they were coded. We conducted the qualitative analysis using Microsoft Word.

## RESULTS

We identified 129 program leadership contacts. Of the 129 survey invitations sent, 4 were returned as undeliverable, and 12 were returned with responses that either the program information was incorrect (i.e., the program was not a fellowship program or did not have a GH component) or the program contact was incorrect. Ten of these responses included corrected program contacts to whom we sent new survey invitations. This resulted in 123 valid invitations sent to contacts at 118 programs, including 52 program contacts in emergency medicine, 25 in family medicine, 16 in pediatrics, 14 in internal medicine, 8 in multidepartmental programs, 6 in obstetrics/gynecology, and 2 in surgery. Forty-five individuals completed the survey, resulting in a response rate of 37%. Responses to individual questions were not required, so the number of responses varied by question and is indicated in the tables, the supplemental figures, and below.

Eighty-four percent (37/44) of respondents were GH fellowship program directors, 2% (1) were associate program directors, and 16% (7) were GH faculty members. One respondent indicated that they did not have a role in GH education (this response ends the survey without asking additional questions). Fifty-two percent (23/44) of respondents were involved with GH fellowships in emergency medicine, 25% (11) were in pediatrics, 16% (7) were in family medicine, 11% (5) were in internal medicine, 2% (1) were in internal medicine–pediatrics, and 2% (1) were in obstetrics and gynecology (Table 1). For subsequent questions where results are reported by specialty, we report the responses from the five respondents who selected both emergency medicine and pediatrics as “pediatrics/emergency medicine,” and we report the response from the one respondent who selected both internal medicine and internal medicine/pediatrics as “pediatrics/internal medicine.”

**Table 1:** Demographics and characteristics of respondents and their global health fellowship programs (*N* = 44 unless otherwise noted)

Demographics and Characteristics	No. (%)
Role in Global Health Education	
Global Health Fellowship Program Director	37 (84)
Global Health Fellowship Associate Program Director	1 (2)
Global Health Faculty Member	7 (16)
Other: Division Director, Division Chief, Vice Chair, Global Health Director, or Director of Global Health Program	5 (11)
Department(s) Housing Global Health Fellowship	
Emergency Medicine	23 (52)
Pediatrics	11 (25)
Family Medicine	7 (16)
Internal Medicine	5 (11)
Internal Medicine/Pediatrics	1 (2)
Obstetrics and Gynecology	1 (2)
Multidepartment	1 (2)
Not Specified	1 (2)
Current Status of Global Health Fellowship Program	
Open and Currently Accepting New Fellows	35 (80)
Open and Currently Not Accepting New Fellows Because of the Pandemic or Other Reasons but Expect to Accept Fellows Again in the Future	8 (18)
Currently Have a Program, but the Program Is Closing	1 (2)
Had a Program, but the Program Is Now Closed	0 (0)
Fellowship Program Size: Number of Approved Fellowship Positions per Year/Enrollment Cycle	
1	20 (45)
2	17 (39)
3	2 (5)
4 or More	5 (11)
Years in Existence of the Global Health Fellowship Program	
<1	1 (2)
1–5	10 (23)
6–10	15 (34)
>10	18 (41)
Duration of the Global Health Fellowship Program in Months, *N* = 43	
<6	0 (0)
6–12	8 (19)
13–24	29 (67)
>24	6 (14)
Additional Global Health Certifications and Degrees Offered	
Master of Public Health	30 (68)
American Society of Tropical Medicine and Hygiene Certificate of Knowledge in Clinical Tropical Medicine and Travelers’ Health	16 (36)
Diploma in Tropical Medicine and Hygiene	14 (32)
Master of Science in Clinical Epidemiology	7 (16)
Other Certification/Degrees: HELP Course, Harvard Humanitarian Response Course, International Emergency Department Leadership Institute Course, MBA, MEd, Graduate Certificate in Global Health, ISTM, Basic Emergency Care Course, Health Emergencies in Large Populations Course, Master of Infectious Disease, MS Clinical and Translational Research, Master of Development Practice, Master of Science and Health Policy	16 (36)

ISTM = International Society of Travel Medicine; MBA = Master of Business Administration; MEd = Master of Education; MS = Master of Science.

### Characteristics of fellowship programs.

Eighty-four percent (37/44) of respondents’ programs had one to two approved fellowship positions per year. The length of most fellowships (67%, 29/43) was 13–24 months. Ninety-one percent (40/44) of respondents indicated that their fellowship offered additional GH clinical certification and degrees. Five percent (2/44) of respondents had accredited/endorsed fellowships, both by SAEM. Most respondents indicated that their programs were relatively long standing, with 75% (33/44) in existence for more than 5 years. Eighty percent (35/44) of respondents indicated that their fellowship program was open and currently accepting new fellows (Table 1).

### GH core content and competencies.

Eighty-nine percent (39/44) of respondents indicated that there is core GH educational content that is important for GH fellows to learn regardless of specialty, with the remaining 11% (5) indicating that they were “unsure.” Fifty-five percent (24/44) of respondents indicated that having some standardized core content and competencies for all GH fellowships was “very” or “extremely” important (Supplemental Figure 1).

### Perceptions of accreditation in general.

Responses to the question of how beneficial some type of accreditation would be for GH fellowships were widely distributed, with 30% (13/43) indicating that it would be “very” or “extremely” beneficial and 21% (9) of respondents indicating that accreditation was “not at all” beneficial (Supplemental Figure 2).

The importance of potential benefits of accreditation varied from 27% (12/44) of respondents indicating that “increased visibility/competitiveness of GH fellowships” was a “very” or “extremely” important benefit to up to 64% (27/42) of respondents indicating that the “ability to contribute to reducing health disparities” was “very” or “extremely” important (Supplemental Figure 3).

When asked what form of accreditation would most likely result in training the most competent GH practitioners, 35% (15/43) of respondents indicated that accreditation is not necessary, 21% (9) selected accreditation from a professional society, and 16% (7) selected ACGME accreditation, with the remaining respondents selecting focused practice or professional certificates (Table 2). We have summarized the reasons given for the form of accreditation selected in Table 2. As we coded the written responses for those that selected “accreditation is not needed” and “other” with codes related to the potential impacts of accreditation, we have reported them with the written responses to the open-ended question about the impacts of ACGME accreditation in the Additional potential impacts of accreditation section below.

**Table 2:** Form of accreditation that global health fellowship leaders indicated would be most likely to result in training the most competent practitioners of global health

Form of Accreditation	All Responses, No. (%)	Responses by Specialty No. (%)		Reasons for Supporting a Specific Type of Accreditation
Accreditation from a Professional Society (e.g., the Academic Pediatric Association)	9 (21)	EM	4 (22)	Would Recognize Global Health as a Distinct Specialty Allows for Specialty-Specific Practices, Competencies, and Goals Professional Societies Are Well Respected
		FM	1 (14)	
		Peds	3 (50)	
		Peds/EM	1 (25)	
		IM	0 (0)	
		Other	0 (0)	
Accreditation Council for Graduate Medical Education	7 (16)	EM	3 (17)	Aligns with Other Subspecialty Fellowships Promotes Standardization with High Standards Allows for Broad Specialty Inclusion
		FM	1 (14)	
		Peds	1 (17)	
		Peds/EM	1 (25)	
		IM	0 (0)	
		Other	1 (25)	
Professional Certificate (e.g., ASTMH CTropMed^®^, DTM&H)	4 (9)	EM	1 (6)	More Flexible and Inclusive Approach Would Create a Shared Body of Knowledge
		FM	1 (14)	
		Peds	0 (0)	
		Peds/EM	0 (0)	
		IM	1 (25)	
		Other	1 (25)	
Focused Practice (by the American Board of Medical Specialties)	2 (5)	EM	2 (11)	Allows for Specialty-Specific Perspectives
		FM	0 (0)	
		Peds	0 (0)	
		Peds/EM	0 (0)	
		IM	0 (0)	
		Other	0 (0)	
I Do Not Think Accreditation Is Necessary	15 (35)	EM	7 (39)	Reported with “Additional Potential Impacts of Accreditation” (Table 3)
		FM	4 (57)	
		Peds	0 (0)	
		Peds/EM	0 (0)	
		IM	2 (50)	
		Other	2 (50)	
Other	6 (14)	EM	1 (6)	Reported with “Additional Potential Impacts of Accreditation” (Table 3)
		FM	0 (0)	
		Peds	2 (33)	
		Peds/EM	2 (50)	
		IM	1 (25)	
		Other	0 (0)	

ASTMH CTropMed^®^ = American Society of Tropical Medicine and Hygiene Certificate of Knowledge in Clinical Tropical Medicine and Travelers’ Health; DTM&H = Diploma in Tropical Medicine and Hygiene; EM = emergency medicine; FM = family medicine; IM = internal medicine; Peds = pediatrics. *N* = 43: EM = 18; FM = 7; Peds = 6; Peds/EM = 4; IM = 4; other = 4 (Peds/IM, obstetrics and gynecology, multidepartment, or not specified).

### Perceptions of ACGME accreditation.

Fifty-two percent (23/44) of respondents either “disagreed” or “strongly disagreed” that there was a need for ACGME accreditation, whereas 25% (11) of respondents “agreed” or “strongly agreed” (Supplemental Figure 4). Seventy-three percent (32/44) of respondents were unsure or did not feel that there would be interest at their institution in an ACMGE-accredited GH fellowship if offered. More than 65% of respondents indicated that the potential loss of flexibility in training fellows, restrictions on funding fellows, additional programmatic requirements, increased administrative burden, and potential increased funding burden on fellows were “very” or “extremely” important barriers to ACGME accreditation (Supplemental Figure 5).

### Additional potential impacts of accreditation.

We found potential positive impacts of accreditation in the written comments, including standardization and formal recognition for trainees. We identified concerns related to increased restrictions, increased burdens, lack of impact, and accreditors as potential negative impacts of accreditation (Table 3). We also found two concerns related to the process of considering accreditation, including the need for more information and broader discussion including international partners.

**Table 3:** Potential positive and negative impacts of accreditation generally and ACGME accreditation specifically

Categories	Codes	Illustrative Quotes
Potential Positive Impacts of Accreditation		
Positive Impacts	Standardization	“I think that accreditation is necessary to standardize training for minimum standards.” General “I think standardization of minimum competencies would be beneficial.” ACGME
	Formal Recognition for Trainees	“[Accreditation would] add some clout to graduates of our training programs.” General
Potential Negative Impacts of Accreditation		
Increased Restrictions	Decrease Individual Program’s Flexibility	“Physicians may contribute to global health equity via a number of career pathways. Their training should be flexible to allow them to adapt their training to match how they will continue to contribute over their careers. I worry about too rigid a training system that will not allow this flexibility between trainees and over time.” General “Global health often includes emergencies—pandemics, refugee crises—that are unplanned. I am concerned about the rigidity of an ACGME-accredited fellowship in global health limiting the ability of a fellow to train in those settings.” ACGME
	Decrease Current Variability/Breadth of Global Health Fellowships	“Many of the fellowships right now have a certain focus each offers to potential applicants and the applicants can choose the type of GH they really want to do—local/global, US-heavy, clinical heavy, academic/research focused, etc. These focuses are also set up based on the assets and resources each program has. Accreditation would take it away.” General “ACGME is very burdensome and not flexible enough to accommodate the various forms a GH fellowship can take.” AGCME
	Limit Which Trainees Can Participate	“A global health fellowship should be open to international trainees from lower-income countries and the training of US and lower-income country trainees should be the same and integrated.” General “ACGME-accredited fellowship is more likely to lead to increased restrictions on who can participate.” ACGME
	Financial	“Asking for ACGME recognition is problematic as ACGME residency programs are supported by CMS dollars. It would be difficult to get CMS funding for work abroad, which I think is a necessary component of a global health fellowship.” “ACGME accreditation would lead to drops in salaries for many global health fellows, which would make it less desirable.”
	International Partnerships	“I also fear ACGME would not be as inclusive to global health partners due to the rigor and structure that are required for all other training programs.”
Increased Burdens	Financial	“[A]ccreditation … would create significant administrative burden, financial burden.” General
	Bureaucratic/Administrative	“Not interested in more red tape.” General “ACGME accreditation brings in an extremely high level of bureaucracy and administrative work.” ACGME
Lack of Impact	Lack of Impact on Recruitment/Employability	“[W]e do not expect [accreditation] to make a difference in recruitment nor job marketability/security after graduation.” General “Currently, I know of few global health employers that require fellowship training of any sort in Global Health. There is therefore little short-term benefit of accreditation to our trainees.” ACGME
	Not Necessarily Needed for Compliance	“Is accreditation needed to ensure adherence to minimum competencies? I’m not totally sure.” ACGME
	Current Fellowships Already Accepted/Valued	“Accreditation is unnecessary—Master’s degree and DTMH are tangible results, and fellowships have been around long enough to gain acceptance.” General
Concerns About Accreditors	Limited Decision-Makers	“[Accreditation] almost always involves a select group of individuals deciding what is important for all.” General
	Lack of Global Health Expertise	“[I]t seems that having the accrediting agency be as close to and well-versed in global health as possible would be an asset, because there are some current issues in global health that I think GME in general are not really engaged in.” General
	Centers Accreditation in the United States/Global North	“[T]here are many potential unintended consequences of picking an accrediting body that is in the global north often controlled by folks who do not practice GH.” General

ACGME = Accreditation Council for Graduate Medical Education; CMS = Centers for Medicare & Medicaid Services; DTMH = Diploma in Tropical Medicine and Hygiene; GH = global health; GME = Graduate Medical Education; US = United States.

## DISCUSSION

As the field of GH continues to develop and mature, considerations of the value of standardized core content, competencies and program requirements, and the benefits of accreditation for GH fellowships become increasingly important. To our knowledge, this is the first multispecialty study to assess the perceptions of United States-based GH fellowship program leaders about core content and accreditation. We found consensus around the existence of important core content for trainees across specialties. Opinions were divided on the need for accreditation, with a slight majority indicating that ACGME accreditation is unnecessary.

We found that most respondents (89%) agree that there is important core content for fellows to learn regardless of specialty, and a slight majority indicated that some standardized core content and competencies are “very” or “extremely” important. This suggests a potential base from which a GH subspecialty could be developed as specified core content and competencies provide the foundation for defining a medical subspecialty. We also found concerns expressed in the written comments about standardization: specifically that standardization could lead to a loss of individual programs’ flexibility to tailor their curriculum to their learners’ interests and goals and that it could decrease the overall breadth and variability of GH fellowships offered. These concerns are consistent with other specialties when considering fellowship accreditation.^32^^–^^34^ They also suggest that developing and implementing core content would require more discussion and consensus building, particularly given the breadth of GH within and between specialties.

Our data show a lack of agreement about whether accreditation would be beneficial and if so, what type of accreditation. Respondents’ differing perspectives on the best approach to accreditation largely centered on two issues: standardization versus flexibility and a multispecialty approach versus a specialty-specific approach. Respondents who indicated that accreditation through professional societies, professional certification, or focused practice would result in training the most competent GH practitioners noted these non-ACGME pathways would allow for specialty-specific practices, competencies, and goals and that they were more flexible approaches. Respondents selecting ACGME accreditation emphasized that it promotes high standards and allows for broad specialty inclusion. Future discussions of accreditation for multispecialty GH fellowships would need to consider how specialty-specific needs could be balanced with standardization across specialties.

In our study, a slight majority of respondents “disagreed” or “strongly disagreed” that ACGME accreditation is needed. This is consistent with a recent survey of GH fellows, which found that 50% of fellows were “not at all” concerned about accreditation by ACGME or other regulating bodies and that 52% did not desire fellowship accreditation.^29^ The perceived barriers to ACGME accreditation identified as “very” and “extremely” important by a substantial majority (>65%) in our study related to increased restrictions, requirements, and burdens. These have been noted with other specialties considering ACGME accreditation.^32^^–^^34^ We also found additional concerns specific to GH fellowships, including financial concerns regarding restrictions on using Centers for Medicare & Medicaid Services funding for international rotations and decreased fellow salaries because of ACGME restrictions. There were also concerns related to the global context of GH, specifically the impact of accreditation on international partnerships, the ability for trainees from low- and middle-income countries (LMICs) to participate in fellowships, and the implications and potential unintended consequences of centering accreditation for GH in the United States. Given the colonial origins of GH and the goal of equity in GH education,^35^ these considerations are especially important. These concerns highlight challenges of ACGME accreditation for GH fellowships because of the international aspects of training and suggest a need for innovative approaches to accreditation to be successful.

Notably, we found variation by specialty regarding ACGME accreditation. Sixty-six percent of respondents in pediatrics “agreed” or “strongly agreed” that ACGME accreditation is needed compared with 11% of respondents from emergency medicine. It is possible that this difference may, in part, reflect pediatric GH educators’ recent successful implementation of accreditation through APA^25^ as this was a precursor to pursuing ACGME accreditation for several pediatric subspecialties. Emergency medicine practitioners may feel, in contrast, that their SAEM endorsement process is sufficient. This suggests that specialties practicing GH may have different interests and needs related to fellowship accreditation that may present challenges for developing a multispecialty fellowship. There are recent successful examples of ACGME-accredited subspecialties accessible from multiple primary specialties, such as clinical informatics,^36^ addiction medicine,^37^ and hospice and palliative medicine.^38^ Some of the approaches and lessons learned from these subspecialties may be relevant to GH fellowship accreditation discussions, particularly approaches to defining a subspecialty^38^ and developing a partnership with an appropriate member board of ABMS.^37^^,^^38^

Our study has several limitations. Limiting our study population to the primary medical specialties may affect the generalizability of our findings to other specialties. Although we identified, to the best of our abilities, all known United States-based GH fellowships in these specialties, there may be programs that we did not identify and therefore, did not include in our study. Our response rate of 37%, although similar to other comparable national physician surveys,^39^ may also limit the generalizability of our findings, with the small number of responses per specialty preventing us from conducting statistical subgroup analysis by specialty.

There are several important next steps in exploring accreditation for United States-based GH fellowships. Further work is needed to better understand the perceptions of other key stakeholders in GH fellowships and accreditation, including colleagues from institutions involved with GH fellowships worldwide, particularly LMIC colleagues and institutions as well as trainees. A broad dialogue with stakeholders should consider key questions suggested from this study, such as how to determine core content, how to fund accredited fellowships, how to allow for broad trainee participation including LMIC participants, how to balance needs for flexibility with standardization, and where to center (geographically and organizationally) an accreditation process. A potential avenue for understanding stakeholder perceptions and consensus building would be dedicated time during relevant meetings, such as at the annual meetings of ASTMH and the Consortium of Universities for Global Health, or to convene a symposium or conference. Further work is also needed to determine whether some of the potential benefits of standardization could be achieved without accreditation. For example, would an approach such as self-reported compliance with consensus external standards work, or is regulatory oversight by an external body through some type of accreditation needed? Additionally, if needed, are there differences in potential benefits depending on the type of accreditation?

## CONCLUSION

In conclusion, this current study suggests that most GH fellowship leaders of United States-based programs feel that there is important core content across specialties. Although some leaders see a potential connection between accreditation and the promotion of standardized content and requirements, our study suggests that there is not a consensus, apart from in pediatrics, that GH fellowship accreditation of any kind should be pursued. A majority disagreed with pursuing ACGME accreditation specifically. More work is needed to better understand broader stakeholder perceptions and build consensus before multispecialty core content and competencies can be defined or accreditation can be pursued. At this time, specialty-specific approaches, such as those currently used by pediatrics and emergency medicine, may be more feasible.

## Supplemental Materials

10.4269/ajtmh.24-0377 Supplemental Materials
