# Supplementary material for: Core Content, Competencies, and Accreditation in US Global Health Fellowships: A Survey of Leaders’ Perspectives
Source: Am J Trop Med Hyg. 2024 Nov 12;112(1):226–33. doi: 10.4269/ajtmh.24-0377 (PMC11720793; doi:10.4269/ajtmh.24-0377)
Supplement: Supplemental Materials [file tpmd240377.SD1.pdf]

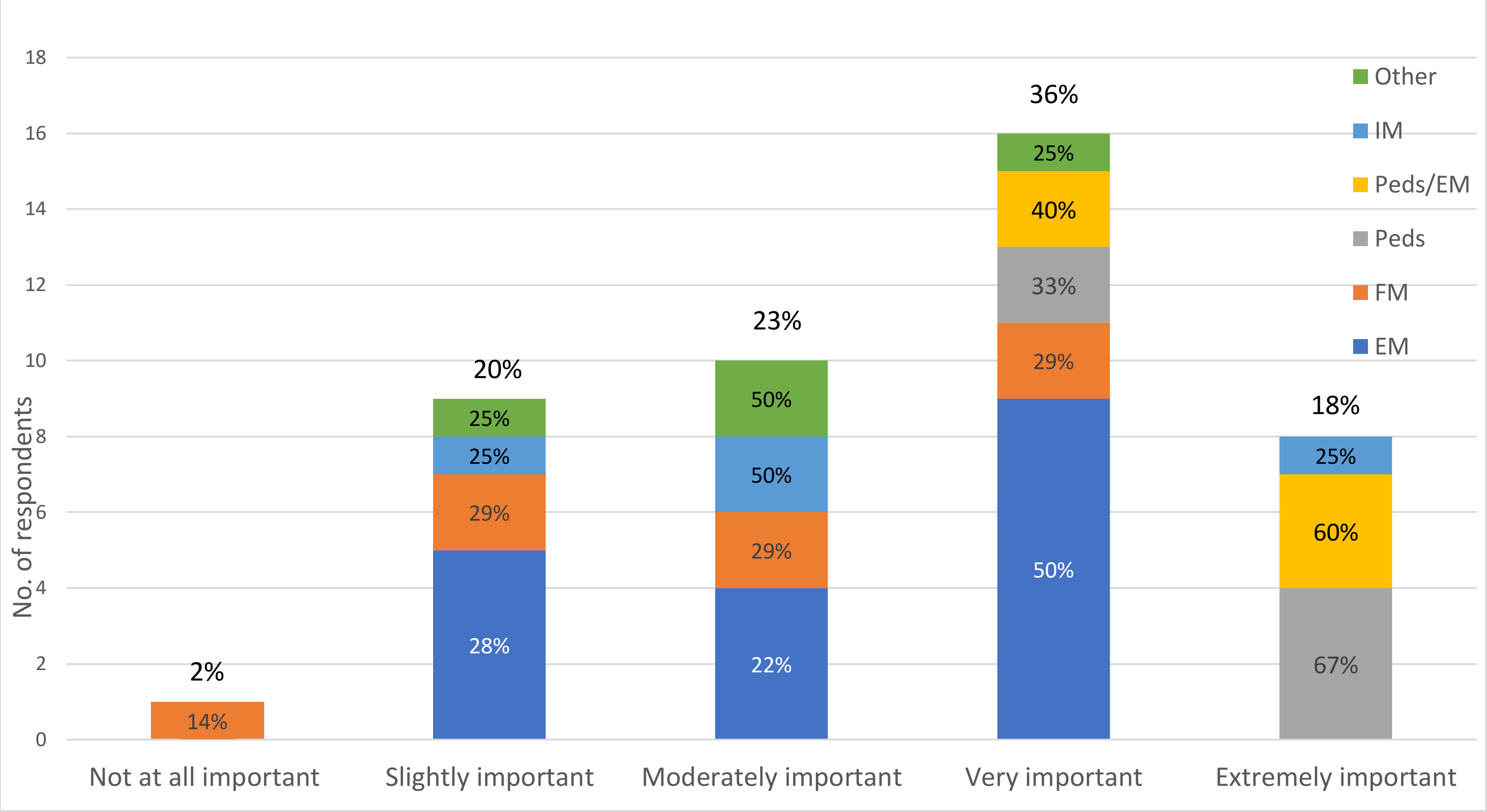

Figure 1. GH fellowship leaders' perceptions of the importance of having some standardized core content and competencies for all GH fellowships. Responses by specialty.

N=44: Emergency Medicine (EM)=18; Family Medicine (FM)=7; Pediatrics (Peds)=6; Peds/EM=5; Internal Medicine (IM)=4; Other=4 (Peds/IM, OB/GYN, Multi-department, Not specified)

% of specialty labeled within column; % across specialties labeled above column

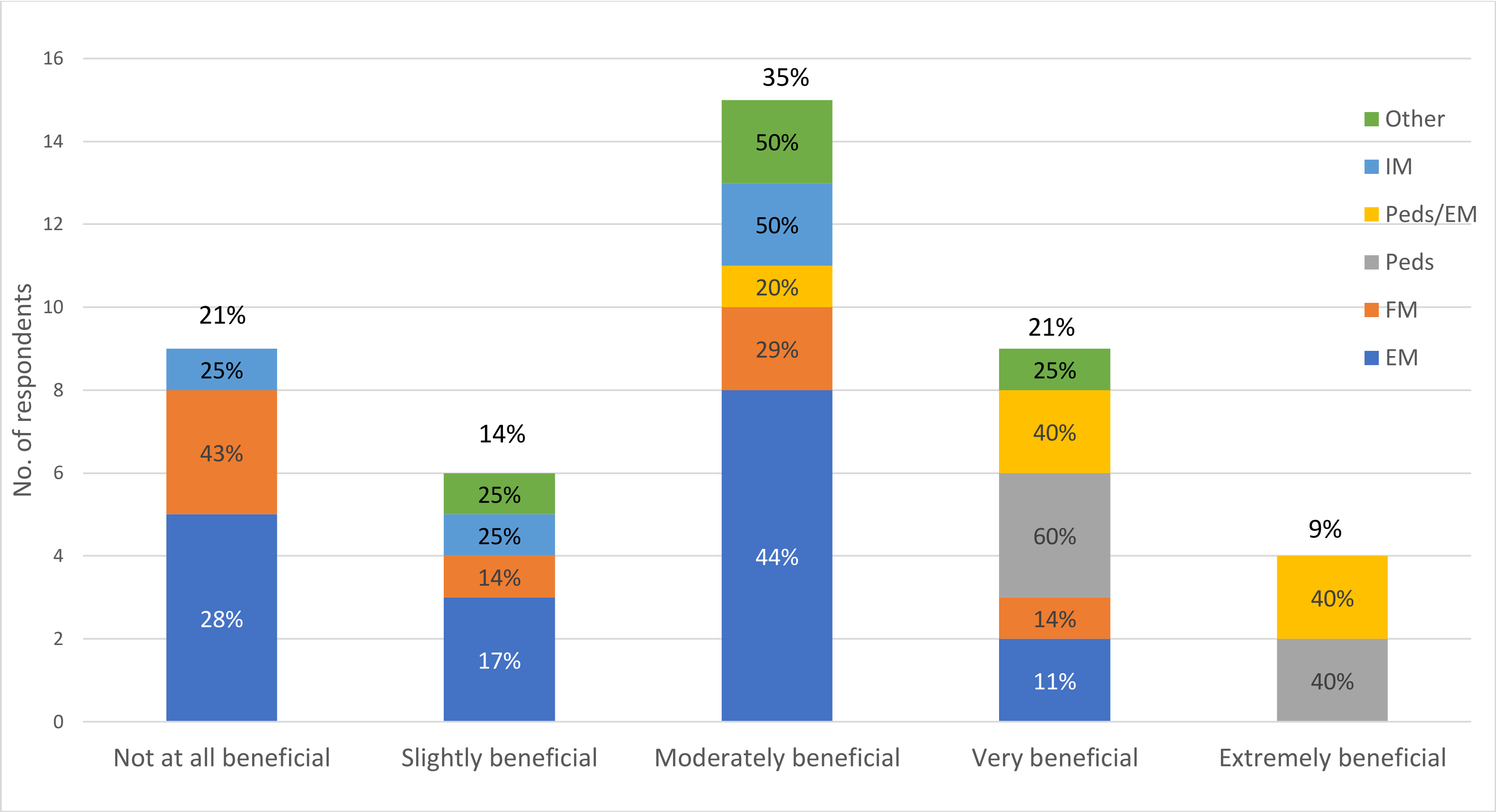

Figure 2. GH fellowship leaders' perceptions of how beneficial some type of accreditation is for GH fellowships.
Responses by specialty
N=43: EM=18; FM=7; Peds=5; Peds/EM=5; IM=4; Other =4 (Peds/IM, OB/GYN, Multi-department, Not specified)
% of specialty labeled within column; % across specialties labeled above column

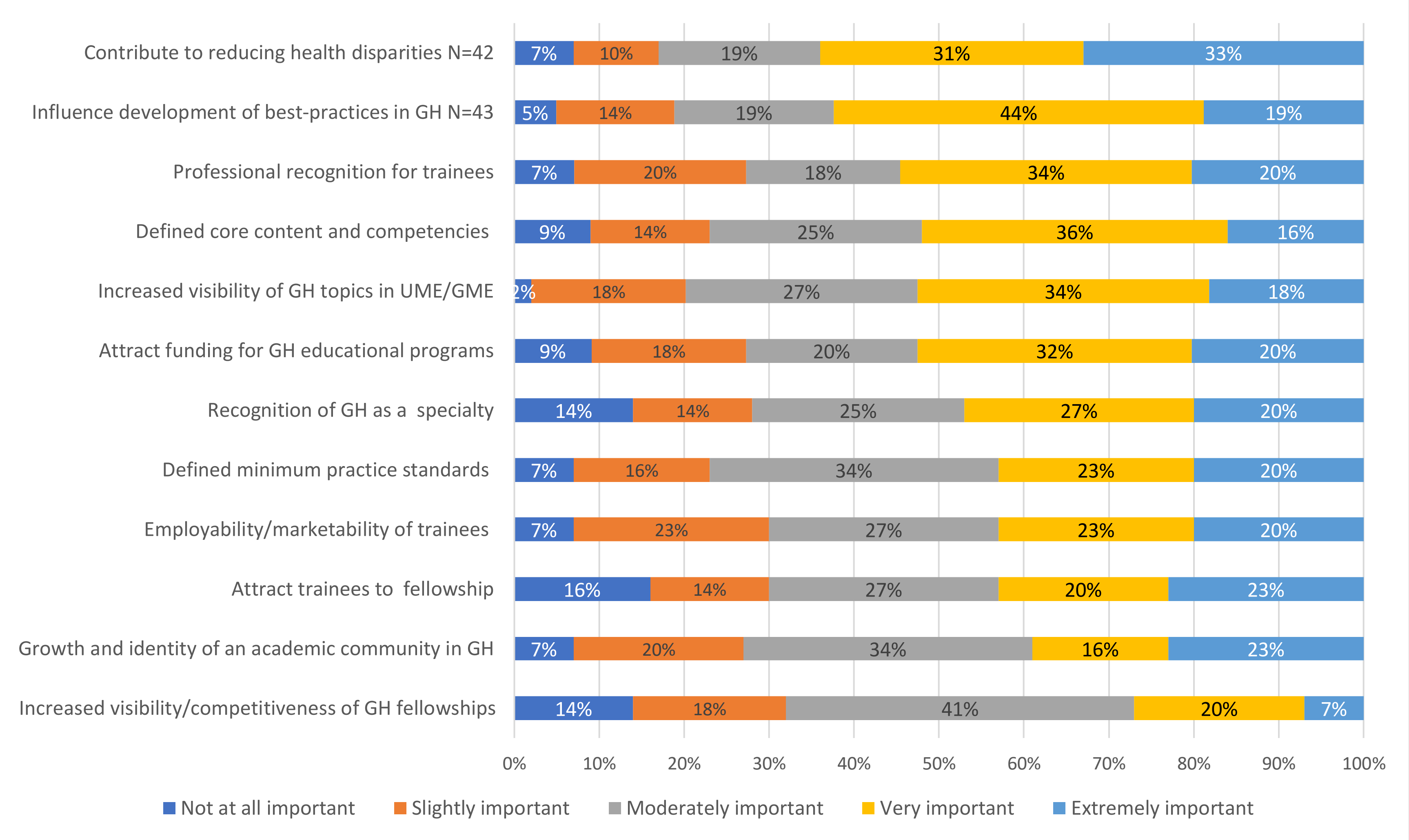

Figure 3. GH fellowship leaders' perceptions of the importance of potential benefits of accreditation. N=44 (unless otherwise noted)

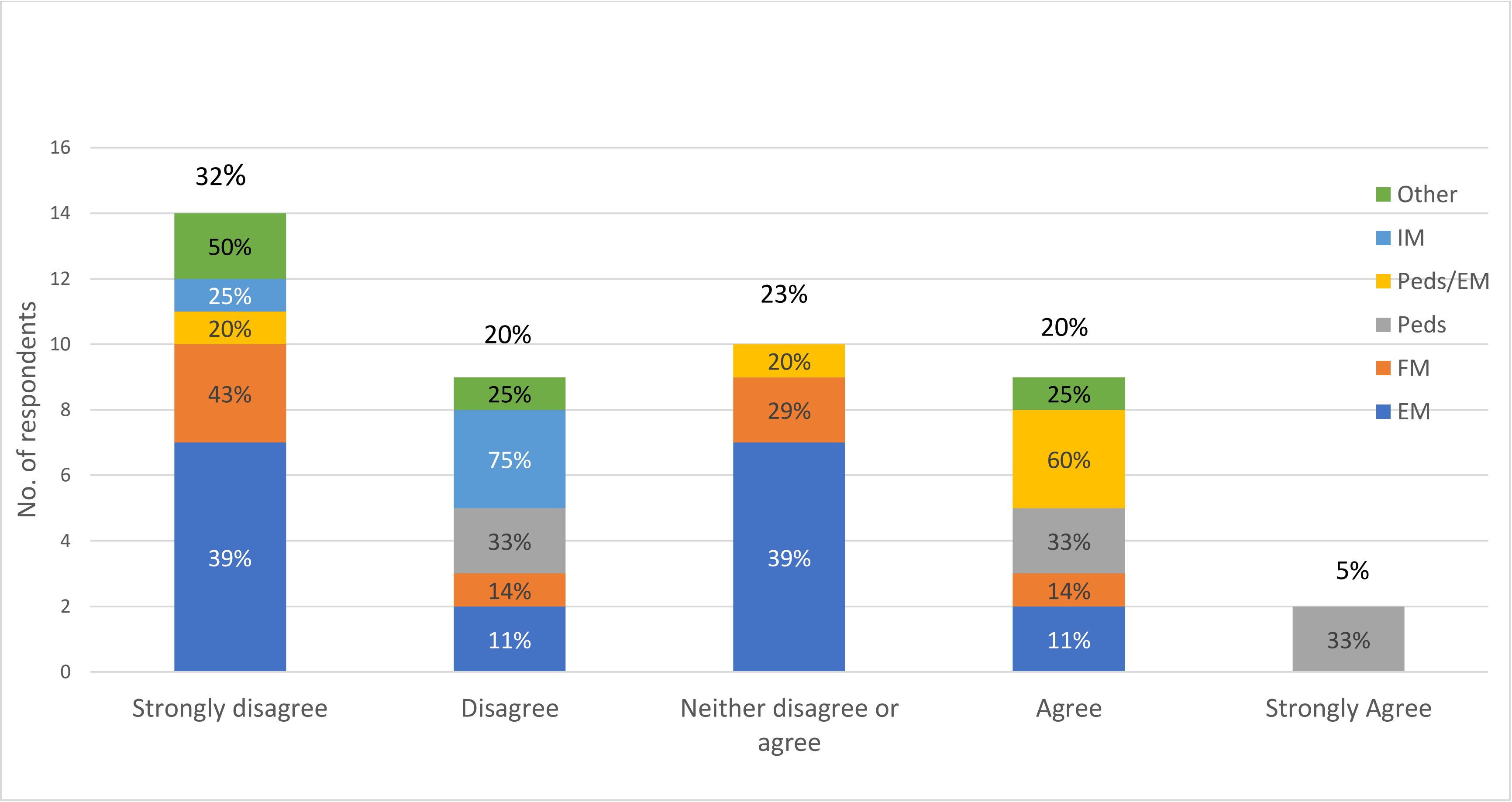

Figure 4. GH fellowship leaders' perceptions of the need for ACGME accreditation for GH fellowships.
Responses by specialty
N=44: EM=18; FM=7; Peds=6; Peds/EM=5; IM=4; Other=4 (Peds/IM, OB/GYN, Multi-department, Not specified)
% of specialty labeled within column; % across specialties labeled above column

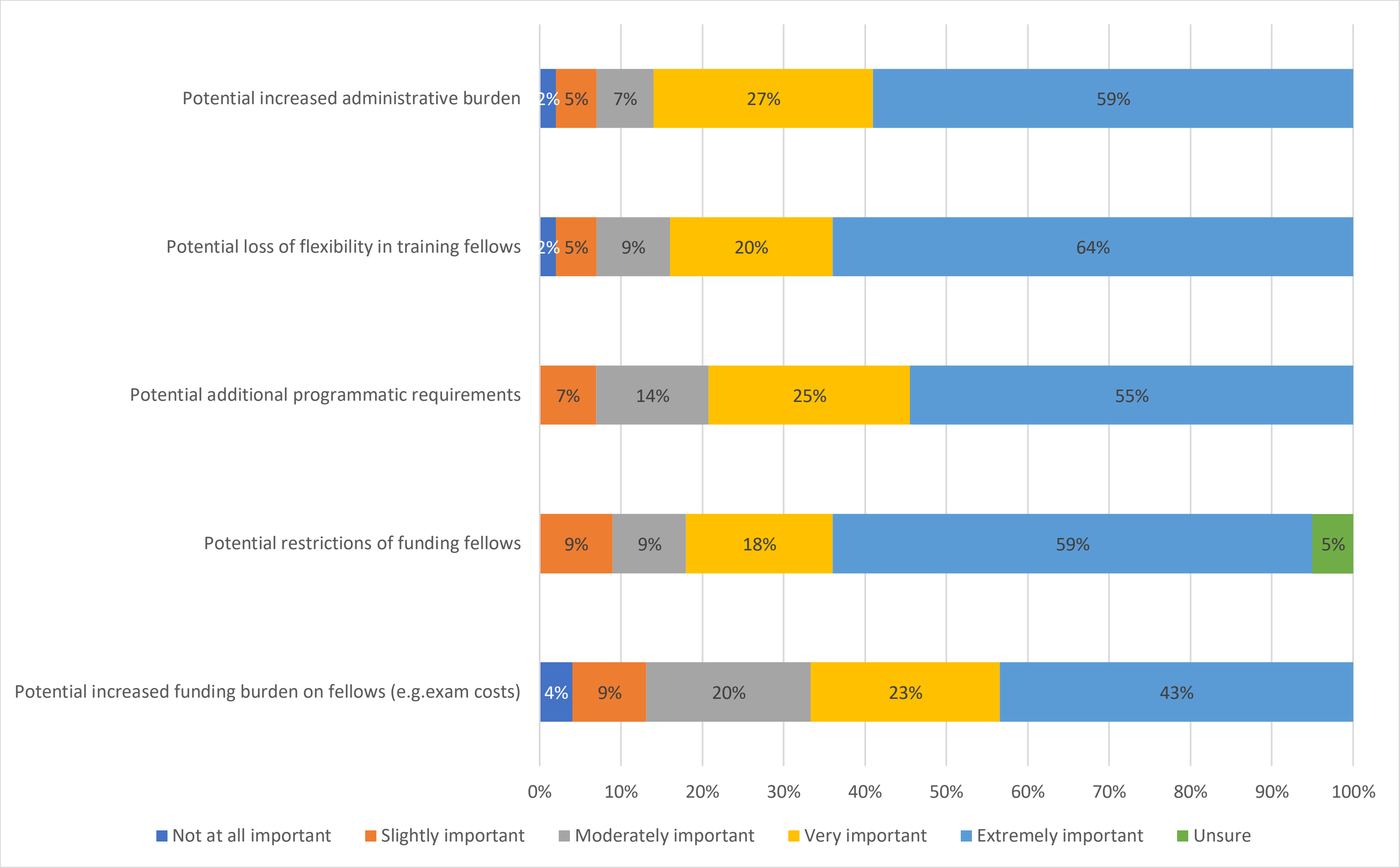

Figure 5. GH fellowship leaders' perceptions of the importance of potential barriers to ACGME accreditation. N=44
